# The science of Durban, AIDS 2016

**DOI:** 10.7448/IAS.20.1.21781

**Published:** 2017-06-30

**Authors:** Chris Beyrer, Olive Shisana, Stefan D. Baral, Koleka Milsana, Kenneth H. Mayer, Anton Pozniak, Bruce D. Walker, Souleman Mboup, Annette H. Sohn, David Serwadda, Helen Rees, Sergii Dvoriak, Mitchell Warren, Safiatou Thiam, Wafaa M. El-Sadr, Xavier Hospital, Owen Ryan, Nicolas Thomson, Linda-Gail Bekker

**Affiliations:** ^a^ Center for Public Health and Human Rights, Johns Hopkins Bloomberg School of Public Health, Baltimore, MD, USA; ^b^ International AIDS Society, Geneva, Switzerland; ^c^ Evidence Based Solutions, Cape Town, South Africa; ^d^ Department of Medicine, University of KwaZulua Natal, Durban, South Africa; ^e^ Department of Medicine, Harvard Medical School, Fenway Community Health Center, Boston, USA; ^f^ Department of Medicine, Chelsea and Westminster Hospital, London, UK; ^g^ Ragon Institute of MGH, MIT, and Harvard, Cambridge, MA, USA; ^h^ Institut de Recherche en Santé, de Surveillance Epidemiologique et de Formations, Dakar, Senegal; ^i^ TREAT Asia, amfAR – The Foundation for AIDS Research, Bangkok, Thailand; ^j^ Department of Epidemiology, Makerere University School of Public Health Kampala, Uganda; ^k^ Wits RHI, University of the Witwatersrand, Johannesburg, South Africa; ^l^ Ukrainian Institute on Public Health Policy, Kiev, Ukraine; ^m^ AVAC, New York, NY, USA; ^n^ Department of Health, National AIDS Council of Senegal, Dakar; ^o^ Mailman School of Public Health, ICAP at Columbia University, New York, NY, USA; ^p^ Department of Health, UNESCO Regional Office in Dakar, Dakar, Senegal; ^q^ Desmond Tutu HIV Research Foundation, University of Cape Town, Cape Town, South Africa

**Keywords:** HIV, treatment, prevention, vaccines, human rights, implementation science

## Abstract

**Introduction**: The science presented at the 21st International AIDS Conference in Durban, South Africa, in July 2016, addressed the state of the field across basic, clinical, prevention, law and policy and implementation science.

**Methods and Results**: The AIDS response has seen remarkable achievements in scientific advances, in translation of those advances into prevention, treatment and care for affected individuals and communities, and in large scale implementation – reaching 18 million people with antiviral therapy by mid-year 2016. Yet incident HIV infections in adults remain stubbornly stable and are increasing in some regions and among adolescents and adults in some key populations, challenging current science, policy and programming. There have been important advances in both preventive vaccines and in cure research, but both areas require ongoing investment and innovation. Clinical research has flourished with new agents, regimens, delivery modes and diagnostics but has been challenged by aging and increasingly complex patient populations, long-term adherence challenges, co-infections and co-morbidities, and unresolved issues in TB management and epidemic control. It is an extraordinary period of innovation in prevention, yet the promise of new tools and combination approaches have yet to deliver epidemic HIV control.

**Conclusions**: Proven interventions, most notably pre-exposure prophylaxis, PrEP, have been limited in rollout and impact. Treatment as prevention has the promise to improve clinical outcomes but remains uncertain as a prevention tool to reduce population-level HIV incidence. The improvement of legal, policy and human rights environments for those most at risk for HIV acquisition and most at risk for lack of access to essential services; sexual and gender minorities, sex workers of all genders, people who inject drugs, and prisoners and detainees remain among the greatest unmet needs in HIV/AIDS. Failure to do better for these individuals and communities could undermine the HIV response.

## Introduction

The 21st International AIDS Conference (IAC), held 18–22 July 2016, in Durban, South Africa, was just the second IAC to be held in Africa, the world’s most HIV-affected continent. The last, in 2000, also in Durban, was a watershed in the history of the AIDS response. AIDS 2000 is now widely acknowledged to have been the convening where a global consensus emerged that antiretroviral therapy (ART) should be made available to all who need it. AIDS 2016, in the second decade of the treatment access era, was hosted by a changed South Africa. Prior denial of the epidemic was replaced by an intensive evidence-informed approach to the HIV response. South Africa is now the country with the largest treatment programme in the world, with some 3.4 million citizens on ART and vigorous international collaborative research and implementation efforts underway across all sectors of HIV and TB investigation [1]. Yet KwaZulu Natal, the provincial host for both AIDS 2000 and AIDS 2016, remains one of the highest burden regions for HIV worldwide, with high and sustained incidence, particularly among adolescent girls and young women [2] and high morbidity and mortality among African adult men and women [3,4]. The enormous achievements of treatment access and daunting reality of ongoing HIV transmission among adults in many settings and populations gave an urgency and intensity to AIDS 2016, to the work undone in HIV, and to the need for sustained scientific investment to achieve the goal of an end to AIDS as a public health threat.

While the IAC serves multiple roles as a global convening for those engaged in the HIV response in social, political and health systems efforts, AIDS 2016 also provided a venue for presentation of an enormous array of scientific achievements and advances.

## Discussion

### Recent advances in basic and translational research

Basic research continues to be of vital importance to two increasingly interrelated goals in HIV, efforts toward sustained remission and HIV cure and toward a preventive vaccine. The new *IAS global scientific strategy: Towards an HIV cure 2016* [5] laid out the challenges of the persistence of HIV viral reservoirs facilitating viral evasion from both host immune responses and current antiretroviral (ARV) drugs. It also detailed strategies to address those reservoirs. Potential new synergies between cancer immunotherapy and HIV vaccines research are energizing this key area of inquiry. The report of the first clinical trial data on the “shock and kill” approach to HIV cure, was, however, a disappointment as all of the patients studied had HIV viral rebound after treatment cessation [6].

Encouraging immunogenicity of a prophylactic HIV vaccine in South African subjects was noted with the prime boost strategy in **HVTN 100** [7]. This study led to a decision to move forward with an efficacy trial, **HVTN 702**, in at-risk adult men and women with this clade C-specific ALVAC-HIV and subtype C gp120 combination antigen. HVTN 702 opened enrolment in November 2016.

### Clinical science

Challenges to long-term retention in care and maintenance of successful viral suppression emerged as issues across several studies and multiple populations: among adolescents, pregnant women and adult men. Treatment adherence issues among adolescents were highlighted in South African national data, where older surviving adolescents had significantly worse viral suppression outcomes than younger adolescents and children. Among 15–19 years olds only 61% were suppressed over time, compared to 71% among children aged 5–9 years [8] In the IMPAACT (International Maternal Pediatric Adolescent AIDS Clinical Trials Network) **PROMISE** study, HIV-positive pregnant women with pre-ART CD4 > 400 cells/mm^3^ were randomized either to stopping or continuing ART post-partum. Among women randomized to extended treatment, 23% had virologic failure at one year. Very few had resistance and 86% were found to have stopped taking medication [9]. The **PROMISE** study also had relatively low uptake after the **START** results were released at IAS Science 2015 in Vancouver. Following those findings, all participants in PROMISE were then offered immediate therapy, but only 66% of women not already on ART chose to start after one counselling session. These women needed more time and input to make the decision to initiate therapy [10].

New treatment approaches hold promise for addressing some of these retention and adherence concerns. The **ARIA** Trial showed superior efficacy of dolutegravir/abacavir/lamivudine (DTG/ABC/3TC) fixed-dose combination compared with ritonavir-boosted atazanavir plus tenofovir disoproxil fumarate/emtricitabine (TDF/FTC) in treatment-naïve women [10] . The dolutegravir-containing regimen was associated with 82% viral suppression at one year, less resistance and lower toxicity [11].

The **PADDLE** study investigated a two-drug combination of dolutegravir and lamivudine (DTG/3TC) in ART-naïve patients [12]. Dual therapy with DTG/lamivudine led to rapid virologic suppression with favourable safety and tolerability outcomes and a 5% failure rate. PADDLE is the first report of a successful dual therapy regimen in ARV-naïve patients after 48 weeks of therapy. Long-acting antiviral studies were also reported, including a study of the persistence of rilpivirine following a single dose of long-acting injection (and cabotegravir) which assessed safety, acceptability and long-term persistence of injected ARVs in the tissues [13]. The **LATTE** Trial investigated cabotegravir + rilpivirine as long-acting intra-muscular(IM)-injected therapy and assessed outcomes at week 48, with both every four-week and every eight-week injection intervals. The every four-week regimen is going forward with efficacy testing, and participants reported high levels of satisfaction with the injectable agents [14].

#### Co-infections

Randomized trials among individuals with HIV-related tuberculosis (TB) have shown significant reductions in deaths when starting ART early. However, PLHIV are still twice as likely to die during TB treatment. The **RAFA** trial showed this mortality could be reduced by giving high-dose rifampicin in combination with standard TB treatment to those with severe immunosuppression [15].

The co-infection plenary highlighted the need to significantly improve TB case finding, treat HIV/TB co-infected patients earlier for their HIV disease, and called for new diagnostics and the need for an improved TB vaccine for eventual elimination [16]. A 2 h molecular test for TB diagnosis and rifampin resistance (GeneXpert MTB/RIF) is becoming routine and could also improve TB control efforts [17].

The **ASTRAL 5** trial investigated a two-drug pan-genotypic regimen for HCV infection (sofosbuvir and velpatasvir) in patients co-infected with HIV-1 and found an overall 95% cure rate, which was only slightly lower for subtype 3 (92%), holding promise for a global treatment strategy for this infection [18].

### Prevention advances

The return of the AIDS Conference to Durban was a reminder of how much progress has been made in bio-behavioural prevention science since the 2000 conference. This progress was detailed in a special theme issue of *The Lancet HIV* focused on prevention and published during the IAC. It has now been definitively demonstrated that early initiation of ART decreases transmission. The **PARTNER** study followed more than 1000 HIV-discordant couples during median follow-up of 1.3 years per couple, and after more than 58,000 episodes of condom-less penile–vaginal or penile–anal sex, there were no HIV transmissions among couples where the primary partner was virally suppressed. The **PARTNER** study followed heterosexual and male same-sex couples [19]. A study of HIV discordant same-sex male couples, **Opposites Attract**, being conducted in Australia, Thailand and Brazil, has similarly not found any HIV transmissions from a primary partner who is virologically suppressed [20].

Multiple studies have now demonstrated the efficacy of oral pre-exposure prophylaxis (PrEP) in adherent, high-risk individuals, with diverse risks for HIV acquisition. There have been more than 50 open label extension studies, demonstration projects and feasibility studies of oral, co-formulated tenofovir-emtricitabine (TDF/FTC), either completed, underway or planned [21]. There are also now multiple countries in North America, Europe, Africa and Latin America that have either approved or are about to approve the use of TDF/FTC for PrEP. Mera et al. reviewed data from 32 completed PrEP demonstration projects carried out in the United States with more than 8400 participants, and seroconversion rates were zero in 17 of the trials [21]. The seroconversion rates overall in the 32 studies were about 1% for men who have sex with men, 0.25% for women at risk for HIV and 2% for transgender women. In studies that collected tenofovir drug levels, seroconversions were associated with low medication adherence [21]. The use of PrEP has increased dramatically in the United States with a more than sevenfold increase between the last quarter of 2012 and the last quarter of 2015. There are estimated to be more than 80,000 individuals in the United States currently receiving a PrEP prescription; however, this represents less than 10% of the individuals that the US Centers for Disease Control and Prevention (CDC) estimates could benefit from PrEP [22]. There also are striking disparities with smaller numbers of women proportionally initiating PrEP in recent years and lower proportion of American Black and Latino individuals on PrEP compared to the rate of new infections in the latter populations in the United States [21].

In order to address the paucity of black men who have sex with men (MSM) on PrEP in the United States, the HIV Prevention Trials Network (HPTN) 073 study evaluated the use of client centred care coordination to assist Black MSM in the uptake and adherence with PrEP [23]. Almost 80% initiated PrEP during the study with good short-term adherence; nonetheless, HIV incidence was 2.9% among those who accepted PrEP. Data were presented from **ATN 113**, which enrolled 15–17-year-old at-risk MSM and offered them either individual or group counselling [24]. Adherence substantially declined after the first three months of the study and the annualized HIV incidence was 6.4%, clearly demonstrating the need for more intensive and youth-specific adherence support. A comparable study conducted in 18–22 year olds (**ATN 110**) offered the same intervention and found an HIV incidence of 3.3% [24]. The data from **ATN 110** and **113** show that development of innovative approaches for PrEP for young MSM that differ from those that have been implemented for older adults is urgently needed, which could involve the use of social media [25].

The final results of the **iPERGAY** study were presented [26] which utilized on-demand PrEP for MSM and involved taking two TDF/FTC within 24 hours prior to sex and a pill a day for 2 days post sexual exposure. In the final report of the study, including its open label extension, the overall efficacy of PrEP was 97%. These findings led the French government to approve the use of on-demand, as well as daily, PrEP. A *post hoc* analysis of the **iPrEX** study suggested that having drug levels consistent with at least four pills a week would provide a very high level of protection [27].

Regarding heterosexual serodiscordant couples in the open-label **PARTNERS Demonstration Project** among 1013 HIV discordant couples in Kenya and Uganda, the infected partner was offered ART irrespective of CD4 count and the HIV-negative partner was offered PrEP [28]. The couples were told that PrEP could be discontinued after six months, once the partner was fully virologically suppressed. Eighty per cent of HIV-positive partners initiated ART, whereas 95% of the HIV-negative partners started PrEP, with a high adherence to both strategies (≥85%). There were only four observed HIV transmissions in the course of the study, with an annualized incidence of 0.2%. This was compared to what might have been expected from historical controls, who would have had an HIV incidence of 4.9% consistent with 95% decreased HIV incidence due to ARV use [28].

These ARV prophylaxis studies have shown that ARVs can protect against HIV transmission or acquisition; however, a high level of adherence is required. Thus, other approaches are being considered, such as long-acting injectable cabotegravir [14] and two HPTN studies – one in MSM in the Americas, and the other among women in sub-Saharan Africa – will be initiated to evaluate the acceptability and efficacy of injectable cabotegravir every eight weeks for prevention of HIV acquisition. Another approach is the use of immunoprophylaxis [7], and the first large-scale studies of the use of broadly neutralizing antibodies administered via intravenous infusion every 8 weeks are underway in the HVTN/HPTN **AMP** (Antibody-Mediated Prevention) studies in North and South America and in sub-Saharan Africa (www.ampstudy.org).

The first study of topical chemoprophylaxis, **CAPRISA 004**, provided the first demonstration that topical (vaginal) tenofovir gel was protective against HIV transmission [29], though two subsequent gel studies, the **VOICE** trial [30] and **FACTS 001** [31], did not demonstrate protection. However, when the data were reanalysed by calculating the protective level when the drug was consistently present in vaginal secretions, protective efficacy in all three gel trials exceeded 50%. This is still not as efficacious as consistently used oral TDF/FTC PrEP, but suggests that topical approaches could be further refined. The Microbicide Trial Network **MTN-020/ASPIRE** (A Study to Prevent Infection with a Ring for Extended Use) and **RING** studies evaluating a dapivirine vaginal ring found an overall efficacy of less than 30% [32,33]. However, when the data were reanalysed and presented in Durban [34], the most adherent participants in the top tercile had a decrease in HIV transmission risk of 92%. Thus topical gels or rings may protect women against HIV, but much of the discussion in Durban focused on how to create demand and enhance medication adherence [35].

The CAPRISA group also presented some data regarding the vaginal microbiome and HIV risk. They found that vaginal *Prevotella bivia* was associated with genital tract inflammation and increased the risk of HIV acquisition 13-fold in women with *Prevotella* in their vaginal microbiome compared to those who did not [36]. The postulated mechanism of action was that *Prevotella* increased lipopolysaccharide biosynthesis, and this could lead to chronic genital tract inflammation. Other studies found that women who had *Lactobacillus*-dominant vaginal microflora who were adherent to topical tenofovir gel were much less likely to be infected with HIV compared to women who were adherent who did not have vaginal *Lactobacilli*. They found that tenofovir seemed to be more rapidly depleted in women who had *Gardenella* present in their vaginal microbiome. Taken together, these studies may suggest that future interventions to increase vaginal microbiological health may be part of comprehensive strategies to decrease HIV transmission in women.

### Social and political research, law, policy and human rights

Highlighting the interconnectedness of marginalization and HIV were data confirming that legal and criminal sanction of key populations and their behaviours resulted in low population size estimates [37]. This is concerning as low population size estimates may erroneously overestimate HIV testing coverage which may exacerbate underfunding of the response among MSM and other key populations. Well-designed and robust key population size estimates are important to inform programming. Concerning data highlighted how rarely these size estimates are used to inform country programmes and that there is the consequent need to build capacity around data utilization in programme design [38].

The exclusion of sexual and gender minority group (LGBT) organizations from the UN High Level Meeting on AIDS in June of 2016 confirmed the ongoing challenges facing civil society groups representing these populations in many countries. But evidence is suggesting that political pressure on these groups is forging new alliances and innovation in the ways that these groups are mobilizing around their work [37]. It was shown that increased civil society space such as the ability to “meet, talk and feel free” was directly associated with an increased civil society ability to influence Global Fund concept notes, which in turn led to enhanced programmatic impact [39].

In support of more empowered civil society, evidence also emerged that programming for social cohesion in a multi-level intervention was able to reduce stigma [40]. But stigma is pervasive in many settings for PLHIV. There is a compelling need to ensure that policies to expand HIV testing and treatment do not exacerbate stigma and do not decrease engagement in HIV services. [11, 41].

The **Rights-Evidence-Action (REAct)** human rights monitoring programme demonstrated how HIV-implementing NGOs could document rights violations (such as physical violence, discrimination, breach of confidentiality or failure to obtain informed consent, among others) in complex and conflict settings while being sensitive to the need to protect the documenters [42]. This was tempered by a large systematic review that examined the evidence for impact of human rights interventions which showed that while impact was possible its evidence base was substantially weakened by the lack of investment in methodologically sound evaluations of human rights interventions [43].

Partnerships were considered core to a more mobilized and engaged community and evidence emerged of the successful impact that researcher-community dialogues could have on recruitment into the phase 2b **HVTN 703/HPTN 081** trials (The AMP study) [44]. Coordinated north–south HIV activist partnerships were increasingly able to ensure that PEPFAR Country Operational Plans align with the needs of the affected population through high-impact watchdogging [45] and the interim evidence of the **New York State Fast Track** plan indicates that mobilizing high-level partnerships across government and community can get cities on track to end their epidemics. Better collaboration and partnership between those working on HIV cure research and the wider community of researchers engaged in HIV prevention and treatment could ensure that cure researchers have greater exposure and engagement in the social and ethical aspects of HIV research. An holistic approach integrating biomedical, social and ethical aspects is needed [46].

Optimizing engagement of the community in cure research through the design of innovative methods to reach large numbers of people through the use of technology showed preliminary success [47]. Evidence of emerging technology and innovation such as researchers collaborating with information technology experts and entrepreneurs in Hackathons to expand access to information for key populations [48] was encouraging. While advances in mobile applications such as geospatial “pinging” to alert users to HIV testing sites offers upside, it was cautioned with the need to ensure technology interfaces really responded to the needs of the target group [49].

Ongoing calls for the decriminalization of sex work, drug use and other repressive laws and policies that impact key populations continued throughout the Durban conference. Findings from the **JHU-Lancet Commission on Drug Policy and Health** reported the impact of repressive legal and policy environments on the expanding epidemics of HIV and HCV among people who inject drugs (PWID) in Central Asia, Eastern Europe and Russia [50]. Integrated care for people with drug dependency, HIV and TB in settings where medication assisted therapy, ART and TB could be co-located was strongly supported by participants in this session.

New analyses presented highlighted that it is possible to advocate for equality of service access within existing repressive legal frameworks. This was highlighted by advocacy for condom access in prisons across Africa [51] and the ability of government and service providers to adapt the service delivery model for methadone adherence treatment in Kenya during the fasting month of Ramadan [52].

A spotlight on prisons was provided by the launch of **The Lancet Special Theme Issue: HIV, Viral Hepatitis, and TB among Prisoners**. The need for the provision of healthcare in closed settings and the principles of equality of access for people caught up in criminal justice systems is forcing the HIV community to adjust its strategies and practical examples emerged of more deliberate and sophisticated engagement towards reform of criminal justice systems [53]. *The Journal of the International AIDS Society* also released a **Special Issue on Police, Law Enforcement and HIV**, which highlighted the need to engage police in the national HIV response. Further evidence emerged of the need to focus on operational and local level police as that is where the greatest opportunity exists to expand police knowledge regarding their role in supporting HIV programmes working with key populations [54].

Evidence continued to build for the impact that increasing social protection and food security has on increasing HIV treatment adherence [55] ensuring that in placing human rights at the centre of the HIV response we need to respond at a holistic and social determinant level.

### Implementation science

The first results of the **SEARCH** trial [56] which reached the UN 90 90 90 targets in East Africa showed that much improved engagement with men is possible through broader health services, including screening for hypertension and diabetes apart from the “test and treat” of HIV. More sobering findings on treatment as prevention (TasP) in high burden settings were presented in the outcomes of the large **ANRS 12249 TasP** trial KwaZulu Natal. This community-randomized trial of home-based HIV testing and universal test and treat found high HIV prevalence, increased viral suppression and overall treatment coverage of 41% of HIV-positive persons, but no impact on community-level HIV incidence [57] . Data from the **CAPRISA** group showed the secondary benefits of improving engagement in care for men in their 20s and 30s to reduce HIV risks for younger women and girls. Specifically, they reported an 8-year median age gap between HIV-positive men and women and much lower ART coverage in men consistent with a systematic review presented at the conference [58] . The **SAPPH-Ire Trial** led by Cowan and colleagues assessed enhanced ART and PrEP uptake among 2722 adult female sex workers in Zimbabwe [59]. The study intervention did not measurably improve viral load outcomes but women in both arms showed good uptake and adherence to interventions in sex-worker-supportive environments.

**LINK4Health**, a cluster-randomized controlled trial by the ICAP group, evaluated the effectiveness of a combination strategy for linkage to and retention in HIV care in Swaziland. The intervention included innovations in point of care CD4 staging, accelerated ART initiation, SMS reminders, and on cash incentives and showed substantial benefits in improved linkage and retention in care [60].

HIV self-testing emerged as an important new tool, with good uptake and good safety, including in some low-resourced settings such as Zambia [61]. Self-testing doubled the rate of regular HIV testing among gay men in Australia [62]. And a social media platform increased MSM engagement in HIV testing in India [63].

Differentiated models of care to better serve the geographically isolated communities affected by HIV were reported by numerous groups. This led to a call from one African villager to “Simplify the way I get ARVs, I am tired of Walking” [64].

## Conclusions and policy recommendations

The policy implications of the science of AIDS 2016 are numerous and will require sustained financing and political will to realize.

Immediate therapy is indicated for all individuals living with HIV infection, but adequacy of human resources, longer term retention in care and adherence and the tolerability and costs of second-line medications are emerging as major challenges. The use of community health workers will be key to expansion of ART and attaining 90–90–90 goals. These goals remain important treatment targets, but whether they are sufficient for epidemic control in high-burden communities and populations remains uncertain.

Implementation science has to focus on new models of care, longer term retention, simplified patient management and stigma reduction. Investigation of new agents that may help address adherence such as long-acting ARVs, injectables and efforts to achieve sustained remission off treatment will be important.

Greater engagement of men in HIV care is an emerging priority given the high premature mortality in this group and the likelihood of onward transmission of the virus among men who do not know their HIV infection status are not on ART. This is true for African men, for PWID in many regions and for MSM globally. The global community is also becoming increasingly aware of the major gaps in prevention for adolescents and young adults, as well as barriers to testing and care that they face and concerns about increasing mortality among those living with HIV. Solutions to access challenges that have worked for older adults are not necessarily applicable to young women and key populations.

Successful management of co-infections in TB and HIV will require health service providers to actively find TB cases using GeneXpert MTB/RIF instead of conventional microscopy and culture and to provide ART to such patients in a timely manner as well as to expand effort for TB-preventive therapy. The key to accessing services will be integration of service through joint planning and monitoring and evaluation [16].

Ongoing HIV incidence in adults and increasing incidence in young key populations are major threats to global control. Now is the time to implement and take to scale enhanced primary prevention. A new research agenda that examines the effect of structural, behavioural and cultural interventions which use HIV incidence as outcomes is urgently needed. These should also be combined with proven biomedical interventions such as PrEP, voluntary medical male circumcision and treatment of genital inflammation.

PrEP is working when adherence is good, and the emerging data on effectiveness in multiple populations at risk mandates an expanded rollout of PreP for serodiscordant couples, including where the infected partner is on ART, for behaviourally at-risk adolescents, sex workers, PWID and MSM.

The world urgently needs a preventive vaccine against different HIV variants and effective approaches for sustained remission off therapy, so the research effort must be supported and sustained.

Ensuring human rights protections, law and policy reform and social justice remains the largest challenge in HIV. This is the area where limited success has been achieved. New advances in science will only benefit those who need them most if services can be provided in safety and with dignity. We succeed or fail dependent on our ability to reach and serve those most in need.
